# Supplementary material for: Resolving the full spectrum of human genome variation using Linked-Reads
Source: Genome Res. 2019 Apr;29(4):635–45. doi: 10.1101/gr.234443.118 (PMC6442396; doi:10.1101/gr.234443.118)
Supplement: Supplemental Material [file supp_gr.234443.118_Supplemental_longranger-2.2.zip › longranger-2.2.tar.bz2!/lr2.2-public/lib/stan/cmdstan/stan/doc/api/html/command_8hpp.html]

Stan: src/stan/common/command.hpp File Reference


|  |  |
| --- | --- |
|  | Stan  2.5.0  probability, sampling & optimization |


- Main Page
- Related Pages
- Namespaces
- Classes
- Files

- File List
- File Members

All Classes Namespaces Files Functions Variables Typedefs Enumerations Enumerator Friends Macros Pages

- src
- stan
- common

Classes |
Namespaces |
Functions

command.hpp File Reference

`#include <fstream>`  
`#include <stdexcept>`  
`#include <boost/date_time/posix_time/posix_time_types.hpp>`  
`#include <boost/math/special_functions/fpclassify.hpp>`  
`#include <boost/random/additive_combine.hpp>`  
`#include <boost/random/uniform_real_distribution.hpp>`  
`#include <stan/version.hpp>`  
`#include <stan/io/cmd_line.hpp>`  
`#include <stan/io/dump.hpp>`  
`#include <stan/io/json.hpp>`  
`#include <stan/io/mcmc_writer.hpp>`  
`#include <stan/gm/arguments/argument_parser.hpp>`  
`#include <stan/gm/arguments/arg_id.hpp>`  
`#include <stan/gm/arguments/arg_data.hpp>`  
`#include <stan/gm/arguments/arg_init.hpp>`  
`#include <stan/gm/arguments/arg_random.hpp>`  
`#include <stan/gm/arguments/arg_output.hpp>`  
`#include <stan/mcmc/fixed_param_sampler.hpp>`  
`#include <stan/mcmc/hmc/static/adapt_unit_e_static_hmc.hpp>`  
`#include <stan/mcmc/hmc/static/adapt_diag_e_static_hmc.hpp>`  
`#include <stan/mcmc/hmc/static/adapt_dense_e_static_hmc.hpp>`  
`#include <stan/mcmc/hmc/nuts/adapt_unit_e_nuts.hpp>`  
`#include <stan/mcmc/hmc/nuts/adapt_diag_e_nuts.hpp>`  
`#include <stan/mcmc/hmc/nuts/adapt_dense_e_nuts.hpp>`  
`#include <stan/model/util.hpp>`  
`#include <stan/optimization/newton.hpp>`  
`#include <stan/optimization/bfgs.hpp>`  
`#include <stan/common/write_iteration_csv.hpp>`  
`#include <stan/common/write_iteration.hpp>`  
`#include <stan/common/write_stan.hpp>`  
`#include <stan/common/write_model.hpp>`  
`#include <stan/common/write_error_msg.hpp>`  
`#include <stan/common/do_print.hpp>`  
`#include <stan/common/do_bfgs_optimize.hpp>`  
`#include <stan/common/print_progress.hpp>`  
`#include <stan/common/run_markov_chain.hpp>`  
`#include <stan/common/warmup.hpp>`  
`#include <stan/common/sample.hpp>`  
`#include <stan/common/init_static_hmc.hpp>`  
`#include <stan/common/init_nuts.hpp>`  
`#include <stan/common/init_adapt.hpp>`  
`#include <stan/common/init_windowed_adapt.hpp>`  
`#include <stan/common/recorder/csv.hpp>`  
`#include <stan/common/recorder/messages.hpp>`  
`#include <stan/common/initialize_state.hpp>`  
`#include <stan/common/context_factory.hpp>`

Go to the source code of this file.

|  |  |
| --- | --- |
| Classes | |
| struct | stan::common::NoOpFunctor |
|  | |

|  |  |
| --- | --- |
| Namespaces | |
|  | stan |
|  | Probability, optimization and sampling library. |
|  | |
|  | stan::common |
|  | |

|  |  |
| --- | --- |
| Functions | |
| template<class Model > | |
| int | stan::common::command (int argc, const char \*argv[]) |
|  | |


---

[ Stan Home Page ]

*© 2011–2014,
Stan Development Team.*
